# Supplementary material for: Collection of cancer-specific data in population-based surveys in low- and middle-income countries: A review of the demographic and health surveys
Source: PLOS Glob Public Health. 2023 Sep 8;3(9):e0002332. doi: 10.1371/journal.pgph.0002332 (PMC10490941; doi:10.1371/journal.pgph.0002332)
Supplement: Attachment — Submitted filename: Response to Reviewers.docx [file pgph.0002332.s001.docx]

Wednesday, 05 April 2023

Dear Editor,

Thank you for reviewing our manuscript titled: **“Collection of cancer-related data in population-based surveys in low- and middle-income countries: a review of the demographic and health surveys”.** We are grateful for the valuable comments provided by the reviewers and believe that addressing them has helped to improve our manuscript. We have revised the manuscript using track changes and present our point-by-point responses to the comments below:

**Reviewer 1**

**Comment:** The authors have done commendable job in examining the cancer-related questions in DHS surveys conducted so far. The research paper is timely and provides useful policy implications for data collection in LMICs, which lack quality data from cancer registries.

**Response:** We thank the reviewer for acknowledging the importance and timeliness of our review.

**Comment:** I have few comments and suggestions for the authors to improve this manuscript.

1. There are few language issues such as " A filtered was ....." in the Methods section.

**Response:** Thank you to drawing our attention to this. It has been corrected (Line 131)

2. "Peaking at 100%..." What exactly is peaking, please clear the sentence a little more.

**Response:** We have clarified by rephrasing the statement as “reaching 100% in 2021” (Line 182)

3. Results Section- There are few grammatical errors or typos
i) Cervical Cancer: …in the past 12 months where. I think it is in "in the past 12 months were".

**Response:** That is correct. We have corrected it to “were” (Line 207)

"In five surveys, respondents were if...."

**Response:** We have added the missing word “asked” in sentence (Line 215)

ii) Breast Cancer: "were asked if they have you ever....

**Response:** Corrected (Line 244)

iii) Prostate Cancer: " if they have you ever heard....

**Response:** Corrected (Line 261)

iv) Other Cancers: " .... professions ever told them they.... It should be " ... professionals ever told them that they ...

**Response:** Corrected (Line 276)

4. DISCUSSION SECTION

.... or may have week. It should be ... or may have weak

**Response:** Corrected (Line 332)

5. Why the authors think few questions would have been missed.

**Response:** Thank you for this opportunity to clarify this limitation. As has been acknowledged, while we used a systematic and comprehensive search strategy with the aim of identifying all relevant surveys and cancer-related questions within surveys, it is possible that our search terms were not perfectly sensitive to pick up all cancer-related questions in surveys asking those questions.

6. It is a suggestion to include the data on cancer incidence or mortality rate using GLOBOCAN 2020 to shed light on whether the surveyed countries have low or high incidence or mortality rates of cancers for which questions were asked in DHS.

**Response:** We appreciate the thoughtful suggestion. We have now added more discussion of the findings in the context of country-specific cancer burden (Lines 316-320)

7. It is a suggestion to include geographical heatmap of the countries in which DHS survey data is available showing geographically where there is no DHS survey or no cancer related questions to the countries with maximum surveys or maximum cancer related questions.

**Response:** We have added a heatmap to illustrate the global distribution of surveys with cancer-related questions by number across countries (Figure 4)

**Reviewer 2**

**Comment:** The Demographic and Health Survey gathers a variety of health-related information. While DHS surveys are not designed for cancer surveillance, they can give valuable information for cancer registries, particularly in LMICs where robust cancer registries are lacking. This review provides a concise summary of how cancer questions have been incorporated into DHS.

**Response:** We appreciate the kind comments. Thank you.

**Comment:** This review is strengthened by the inclusion of countries from different continents and multiple years.

**Response:** Thank you.

This article is well-written and has excellent word flow.

**Response:** Thank you.

**Minor comments:**

1. On Page 7: The sentence starting '"Ultimately, findings......would fit in the discussion section.

**Response:** We agree that the statement could fit in the discussion section. Our intention was to use it to bolster the review’s rationale. We have now rephrased it (Lines 91-93) to distinguish it from the more detailed discussion of the implications of the review’s findings for future DHS and similar population-based health surveys in the discussion section (from 301 onward)

2. The method section is long, would benefit from subsection e.g Study design, DHS survey structure, Search Strategy and data collection, Data analysis.

**Response:** We have introduced sub-headings in the methods section as suggested. Thank you.

3.Minor grammatical errors to correct on page 16 , were vs where,..In five surveys, respondents were *Asked* .Only five surverys included questions on *cervical* cancer treatment.... page 17...if they have ever heard.....

**Response:** We have corrected all typos and syntax issues identified.
