# Supplementary material for: Collection of cancer-specific data in population-based surveys in low- and middle-income countries: A review of the demographic and health surveys
Source: PLOS Glob Public Health. 2023 Sep 8;3(9):e0002332. doi: 10.1371/journal.pgph.0002332 (PMC10490941; doi:10.1371/journal.pgph.0002332)
Supplement: Attachment — Submitted filename: Response to reviewers_PGPH-D-22-01991R1.docx [file pgph.0002332.s002.docx]

Thursday, 27 July 2023

Dear Editor,

Thank you for reviewing our manuscript titled: **“Collection of cancer-specific data in population-based surveys in low- and middle-income countries: a review of the demographic and health surveys”.** We are grateful for the additional comments provided by the reviewers and believe that addressing them has helped to improve our manuscript. We have revised the manuscript using track changes and present our point-by-point responses to the comments below:

**Reviewer 2**

**Comment:** All my comments have been responded to.

**Response:** We thank the reviewer again for the helpful comments and are glad that we addressed those satisfactorily.

**Reviewer 3**

**Comment:** The paper presents an interesting description of cancer related data being collected in DHS globally. DHS do represent a potentially valuable source for NCD surveillance data including on risk factors, screening and treatment. An evaluation is therefore worthwhile.

**Response:** We appreciate this acknowledgement of the value of our manuscript.

**Comments:** My main comment would be related to a clear definition of “cancer -related data”. It has not been clearly defined. This being the main outcome variable, needs to be defined. For example cancer related risk factors are covered in the paper, as a peripheral issue. Number of sexual partners is a known behavioural risk factor for cervical cancer, oral pill use has implications for cancer. Household fuel use also fits the bill as a risk factor for cancer and is usually collected in DHS.

**Response:** We have clarified that for the purposes of our assessment, cancer-specific questions refer to those relating to specific aspects of cancer, such as survey participants’ knowledge and awareness of particular cancers; prevalence of commonly occurring cancers; and awareness, practices and trends relating to cancer screening, diagnosis and treatment. (Lines 91-94). We have modified the phrase ‘cancer-related’ in the title and in the manuscript to ‘cancer-specific’ to reflect this specific focus. We have also added that, as a secondary objective, our assessment aimed to quantify and characterise questions specific to alcohol consumption and tobacco use, which are known risk factors of cancer (Lines 94-96). Furthermore, we have added an acknowledgement that our review did not comprehensively explore cancer risk factors beyond alcohol and tobacco use (Lines 394-396) We have noted that a more comprehensive assessment will require a broader objective and search strategy, hence beyond the scope of the current work, while acknowledging that this limitation is an opportunity for future research on the topic to consider (Lines 396-397)

**Comment:** Search strategy does not list them though (line 150) says in the third step it was included? Not sure how anything that is not included in first step can be included in third step.
Either the title and scope of the paper should be changed, or the definition revised. Either way a clear definition is a MUST.

**Response:** As mentioned earlier, alcohol and tobacco use were explored as a secondary objective, given that they are well known risk factors of cancer (Lines 94-96). To identify surveys with questions specific to alcohol consumption and tobacco use, we used a separate search strategy different from that described for the primary objective (described in steps 1 -3 of the search strategy). For the secondary objective, we applied the DHS database’s ‘search by survey characteristics’ search function to identify all surveys in which alcohol and tobacco use questions were asked (irrespective of whether they included cancer-specific questions as defined for the primary objective) since inception of the DHS. This has been clarified (Lines 153-156)

**Comment:** Table 1 can be revised to leave out the countries that do not have any survey on cancer related data. These countries can be added as a legend (with years of survey in brackets) to make the table more reader friendly. Else it is made as a supplementary table. Figure 3 (formerly Figure 4) provides the same information.

**Response:** The essence of Table 1 is to highlight countries where cancer-specific questions have been included in surveys as well as countries where such questions have not been included in surveys conducted. In our view, it is important to draw this contrast, to highlight the ‘where’ and ‘where not’ to the reader. Figure 4 presents a visual summary with the aid of a geospatial heatmap to support the information presented in Table 1.

**Comment:** Figure 2 & 3 can be combined and rather than individual years can be given in blocks of 3 years. It gives a wrong impression when the authors say that in 2021 100% of the surveys asked cancer questions as the number of surveys is 2 and in the previous two years (2019-20), it was quite low. It can be expressed either as numbers or percentages. The figure was not clear in black and white.

**Response:** We appreciate this suggestion. We have merged the information in former Figures 2 and 3 into one figure (new Figure 2).

**Comment:** It is not clear how the authors suggestion of linking RF questions with cancer related section (line 362-366). Authors need to explain this more.

**Response:** We have clarified what me meant, while providing specific examples**.** (Lines 367-372)

**Comment:** 336-338. I would say that surveys provide an opportunity to educate people as the population covered is miniscule as compared to the whole population.

**Response:** We have added that while surveys may provide opportunities for informing and educating people on common cancers, survey participants represent a small proportion of the reference populations. However, including cancer-specific information and questions in surveys may contribute to efforts to increase public knowledge and awareness of common cancers (Lines 337-342)
